# Nine glycolysis-related gene signature predicting the survival of patients with endometrial adenocarcinoma

**DOI:** 10.1186/s12935-020-01264-1

**Published:** 2020-05-24

**Authors:** JinHui Liu, SiYue Li, Gao Feng, HuangYang Meng, SiPei Nie, Rui Sun, Jing Yang, WenJun Cheng

**Affiliations:** 1 Department of Gynecology, The First Affiliated Hospital of Nanjing Medical University, 300 Guangzhou Road, Nanjing, 210029 Jiangsu China; 2 Department of Orthopedic Surgery, The First Affiliated Hospital of Nanjing Medical University, Nanjing, Jiangsu China

**Keywords:** Endometrial cancer, Glycolysis, Prognostic model, TCGA, GSEA

## Abstract

**Background:**

Endometrial cancer is the fourth most common cancer in women. The death rate for endometrial cancer has increased. Glycolysis of cellular respiration is a complex reaction and is the first step in most carbohydrate catabolism, which was proved to participate in tumors.

**Methods:**

We analyzed the sample data of over 500 patients from TCGA database. The bioinformatic analysis included GSEA, cox and lasso regression analysis to select prognostic genes, as well as construction of a prognostic model and a nomogram for OS evaluation. The immunohistochemistry staining, survival analysis and expression level validation were also performed. Maftools package was for mutation analysis. GSEA identified Glycolysis was the most related pathway to EC. qRT-PCR verified the expression level of hub gene in clinical samples.

**Results:**

According to the prognostic model using the train set, 9 glycolysis-related genes including B3GALT6, PAM, LCT, GMPPB, GLCE, DCN, CAPN5, GYS2 and FBP2 were identified as prognosis-related genes. Based on nine gene signature, the EC patients could be classified into high and low risk subgroups, and patients with high risk score showed shorter survival time. Time-dependent ROC analysis and Cox regression suggested that the risk score predicted EC prognosis accurately and independently. Analysis of test and train sets yielded consistent results A nomogram which incorporated the 9-mRNA signature and clinical features was also built for prognostic prediction. Immunohistochemistry staining and TCGA validation showed that expression levels of these genes do differ between EC and normal tissue samples. GSEA revealed that the samples of the low-risk group were mainly concentrated on Bile Acid Metabolism. Patients in the low-risk group displayed obvious mutation signatures compared with those in the high-risk group. The expression levels of B3GALT6, DCN, FBP2 and GYS2 are lower in tumor samples and higher in normal tissue samples. The expression of CAPN5 and LCT in clinical sample tissues is just the opposite.

**Conclusion:**

This study found that the Glycolysis pathway is associated with EC and screened for hub genes on the Glycolysis pathway, which may serve as new target for the treatment of EC.

## Background

Endometrial cancer is a kind of female malignancy. In female tumors, EC ranked fourth. In 2015, the American Cancer Society (ACS) predicted that the number of new cases of EC was 54,870, of which the number of patients died was 10,170. This means that in the past 20 years, the fatality rate of EC has increased by more than 100%. The average age of patients at diagnosis is 63. Among them, 90% of patients are over 50 years old, and only 20% of patients can get diagnosed before they menopause [1]. Though quite a lot of research have been conducted. EC is not amenable to screening, hence needs to be effectively managed once the diagnosis is made [2–5]. Glycolysis of cellular respiration is a complex reaction and is the first step in most carbohydrate catabolism. Most of the glycolysis occurs in the cytoplasm. It does not utilize any molecular oxygen to react. It is a special metabolic pathway. Increased glycolysis is the main source of energy supply in cancer cells that use this metabolic pathway for ATP generation. Increased glycolysis can produce ATP for cancer cells, becoming the main energy source for cancer cell growth and metabolism. Hence Altered energy metabolism was seen to be “hallmarks of cancer” [6]. Research by Ganapathy-Kanniappan et al. Showed that Tumor glycolysis working as a target to treat cancer was very promising [7]. They also found that tumor glycolysis is closely related to immune evasion in cancer, which might be a brand new therapeutic opportunities [8]. Akins et al. found that Inhibition of glycolysis can fight tumors [9]. Li et al. also found that glycolysis can be used as a new target for tumor therapy [10]. Qin et al. found that glycolysis can regulate metastasis of gastric cancer cells [11]. Feinberg et al. also demonstrated that glycolysis was involved in the metabolism of lung cancer [12]. This study focused on the relationship between Glycolysis and endometrial cancer treatment, screening appropriate targets, and opening up new ideas for the treatment of EC.

## Material and method

### Source of obtaining data

TCGA provided mRNA data and the corresponding EC clinical information [13], which was proceeded on platform Illumina HiSeq RNA‐seq [14], containing 552 EC patient samples and 35 normal tissues. We analyzed all EC patients with complete follow-up information. After integrating clinical information, 520 samples were obtained. These samples were classified into the training cohort randomly (n = 260), the testing cohort (n = 260). The training cohort was used for prognostic model construction, while the testing cohort and entire cohort were chosen for validation.

### Gene set enrichment analysis (GSEA)

GSEA (http://software.broadinstitute.org/gsea/index.jsp) [15] was used to determine the Molecular Signatures Database (MSigDB) provided hallmark gene sets in order to predict biological processes between the healthy samples and EC samples. The *P*-value < 0.05 and FDR (false discovery rate) < 0.01 were set as the cutoff.

### Identification of prognostic genes and their characteristics

Univariate regression analysis, Lasso analysis and multivariate regression analysis were applied to explore the correlation between expression levels of glycolysis-related genes and patients’ overall survival (OS). In the univariate Cox regression analysis, gene was seen to be a candidate prognostic gene when *P*-value was < 0.05. Lasso‐penalized and multivariate analysis were next performed for further screening. Hazard ratios (HRs) and regression coefficient were calculated for each gene, and the satisfactory mRNAs were ultimately included. The gene alteration type and frequency, as well as the most frequently altered neighbor genes of satisfactory genes were exhibited by the cBioPortal (cBio Cancer Genomics Portal) tool [16].

### Construction of the gene‐related prognostic model

The prognostic risk-score model for outcomes prediction of EC patients was the combination of each optimal prognostic mRNA expression level multiplying relative regression coefficient weight calculated from the multivariate model according to the following way:

$${\text{Risk Score}}\left( {\text{patient}} \right) = \mathop \sum \limits_{i} {\text{Coefficient}}\left( {{\text{mRNA}}_{i} } \right) \times {\text{Expression}}\left( {{\text{mRNA}}_{i} } \right)$$

All patients from the training cohort were divided into high- and low-risk groups on the basis of the median risk score. The Kaplan–Meier survival curves of both groups were plotted and the ROC (receiver operating characteristic) curve for OS prediction was present to assess the sensitivity and specificity of the model [17]. Cox multivariate analysis regarding several clinicopathological features of EC patients were also performed to exam the independency of the prognostic model without clinical characters.

### Validation of the efficacy of the prognostic risk model

By comparing the testing cohort and entire cohort patient’s risk score with the cut-off value calculated from the training cohort, each patient was categorized as the high-risk or low-risk group. Time-dependent ROC, Kaplan–Meier curve and cox multivariate analysis were also performed. Meanwhile, the stratification analysis was operated based on clinicopathological features.

### Validation of the hub genes

A nomogram and calibrate curve was built by the “rms” package on R. The correctness was examined to check the consistency index between actual observation frequency and predicted probability. Then, we presented the predicted and observed results in the calibration curve to visualize the performance of the nomogram. And the 45° line represents the best prediction. TCGA data was also used so as to validate the glycolysis-related genes expression level between EC and normal samples. And the immunohistochemistry staining of both the normal and EC samples were downloaded from the Human Protein Atlas database (https://www.proteinatlas.org/). Survival analysis was also conducted for hub genes using “survminer” R package and “survival” R package.EC samples from TCGA were divided into two groups based on each hub gene’s best-separation cut-off value to plot the Kaplan–Meier (K-M) survival curves.

### Mutation analysis

The mutation data were processed and visualized by R package maftools.

### Preparation of endometrial cancer clinical tissue samples and normal endometrial tissue samples

Throughout the study, we signed an informed consent form for tissue sample acquisition and analysis with each patient, which was approved by the Institutional Review Committee of Nanjing Medical University. After removing part of the tissues of patients with endometrial cancer, we immediately frozen them and stored them at – 80 °C until they were taken out. From June 2019 to January 2020, the Obstetrics and Gynecology Department of the First Affiliated Hospital of Nanjing Medical University obtained tissue samples from patients with informed consent, including a total of 10 clinical tissue samples of endometrial cancer and 10 clinical cases of normal endometrium Tissue samples.

### Total RNA extraction, reverse transcription and real-time quantitative RT-PCR (qRT-PCR) analysis

We used TRizol reagent (Thermo Fisher Scientific, Waltham, MA, USA) to extract total RNA from tissue samples and Agilent Bioanalyzer 2100 (Agilent Technologies, Santa Clara, CA, USA) with RNA 6000 Nano kit to evaluate the integrity of extracted RNA. We used a high-capacity cDNA reverse transcription kit (Thermo Fisher Scientific) to react with the extracted RNA to synthesize single-stranded complementary DNA from RNA, and then used the SYBR Green PCR kit (Thermo Fisher Scientific) for real-time quantification. Record the cycle threshold (Ct) of each gene. The relative expression of the target gene was calculated using the 2^−ΔΔCt^ method (ΔCt = Cttarget gene-in vitro control). All program steps of real-time quantitative RT-PCR (qRT-PCR) are performed in accordance with the instructions provided by the manufacturer. See Additional file 1: Table S1 for primer sequence.

## Result

### Functional pathway screening using GSEA

**Fig. 1:**
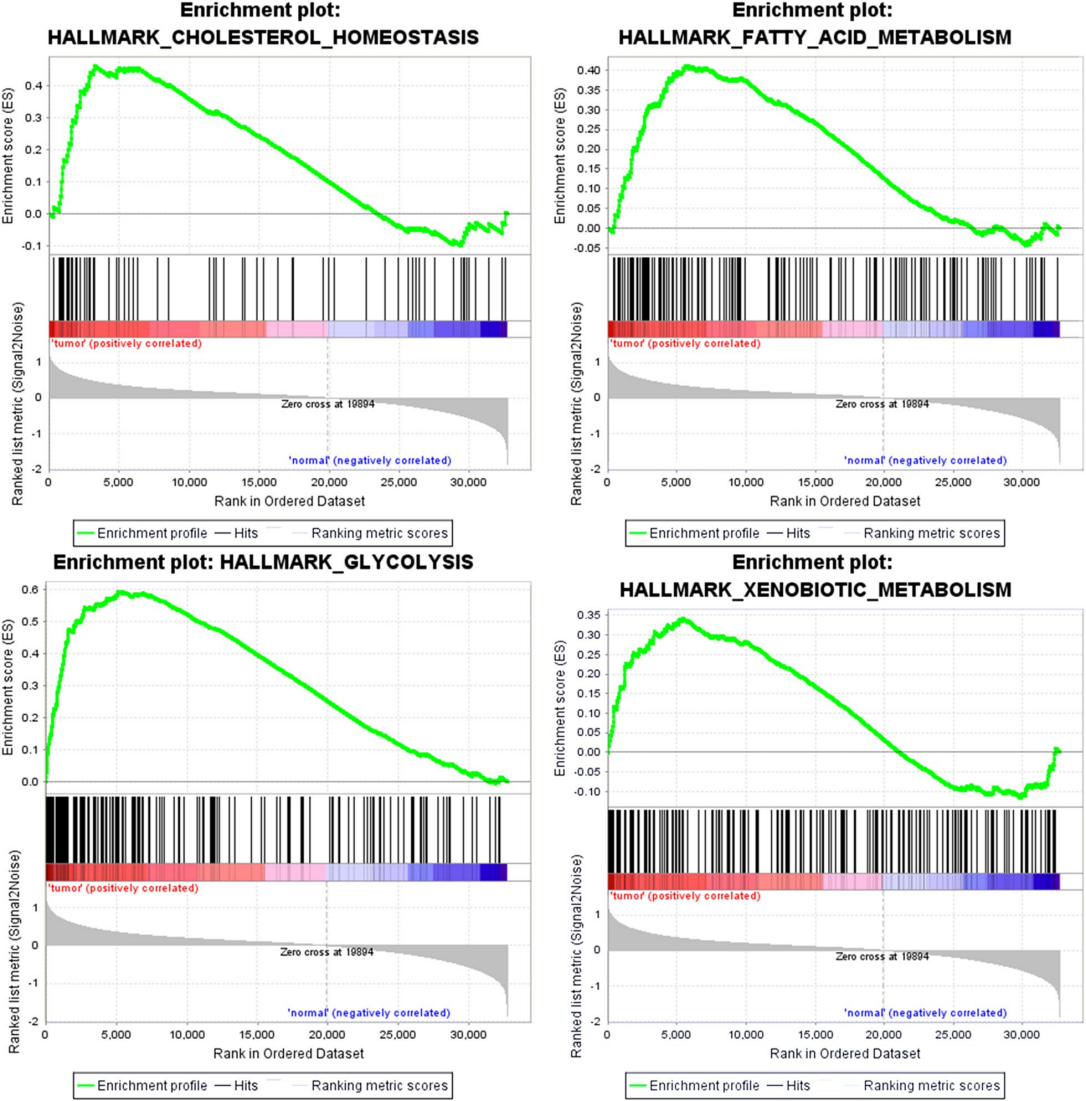
GSEA identified that four gene sets were significantly enriched including glycolysis, cholesterol homeostasis, fatty acid metabolism and xenobiotic metabolism

Clinical features information of a total of 587 samples including 552 EC and 35 healthy samples were achieved from the TCGA. Based on the mentioned data, GSEA indicated that whether the identified gene sets showed significant differences between EC and adjacent healthy tissues. And we found that these genes are significantly enriched in glycolysis, cholesterol homeostasis, fatty acid metabolism and xenobiotic metabolism. Glycolysis was shown to be the most relevant pathway (Fig. 1).

### Establishment of glycolysis-related genes and EC prognosis models

**Fig. 2:**
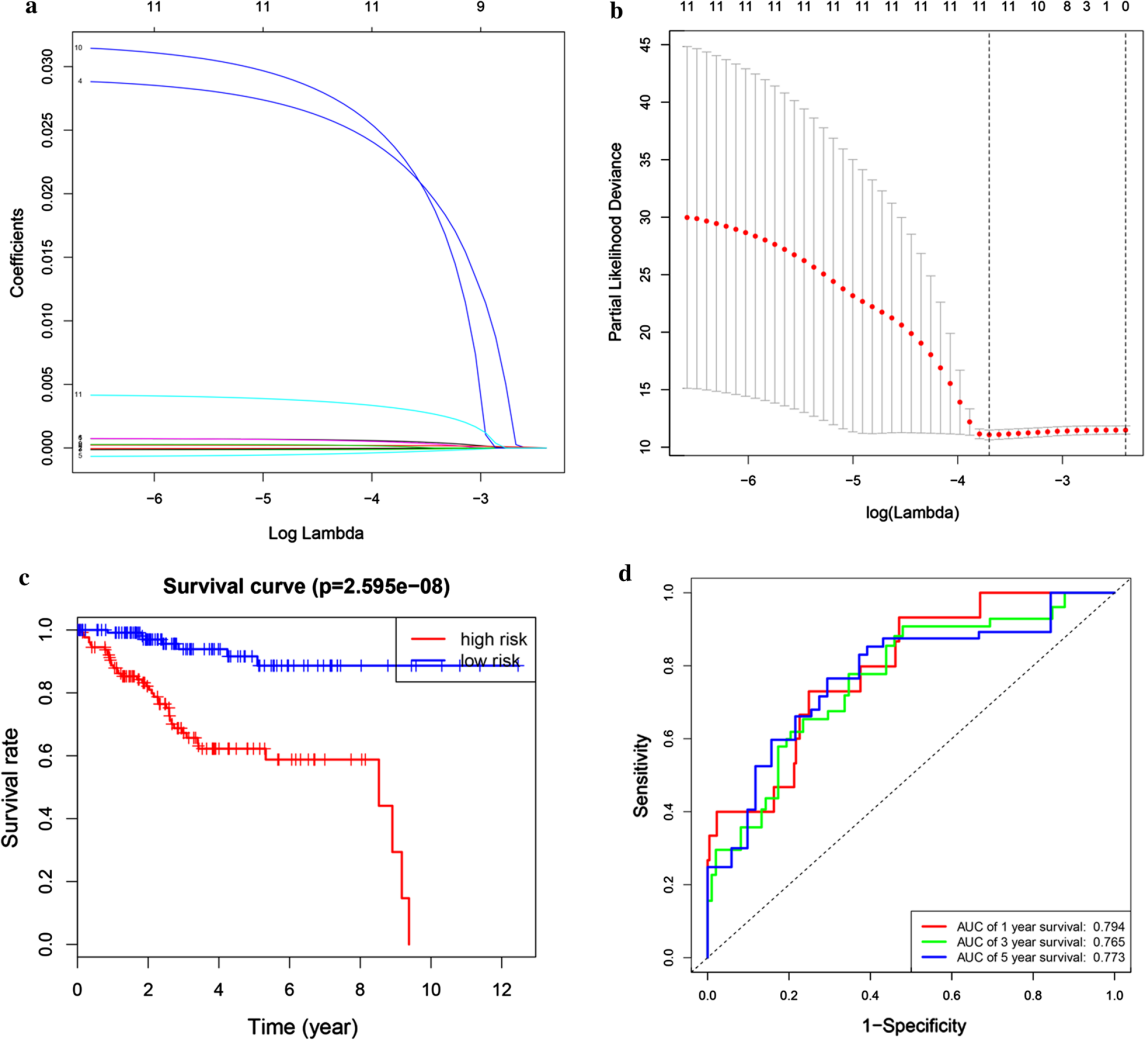
Prognostic model of the training cohort. **a**, **b** The coefficients calculated by LASSO. **c** Kaplan–Meier survival analysis of the low- and high-risk group patients in the training cohort. **d** ROC curve analysis according to the 1, 3, 5-year survival of the area under the AUC value

Firstly we integrated mRNA expression profiles and clinical information so as to screen out 520 EC samples. We analyzed 520 EC samples and found a total of 179 participating genes on the Glycolysis pathway in order to research the relationship between Glycolysis and the prognosis of EC patients. Then we randomly selected 260 samples as training cohort and built a prognostic model for 260 samples. Univariate Cox regression analysis screened out 11 genes with the cutoff of *P *< 0.05. These prognosis-related Glycolysis genes were further analyzed with the least absolute shrinkage and selection operator (LASSO) Cox regression algorithm (Fig. 2a, b). Then multivariate Cox proportional hazards regression analysis built the risk signature. We constructed prognostic models and the risk scores were calculated. Nine genes including B3GALT6, PAM, LCT, GMPPB, GLCE, DCN, CAPN5, GYS2 and FBP2 were identified as prognosis-related genes. The risk score come out as the followed: 0.000755345* B3GALT6-5.19E−05* PAM + 0.029807032* LCT-0.000708518* GMPPB + 0.000784398* GLCE-0.00015091* DCN + 0.000258397* CAPN5 + 0.031956259* GYS2 + 0.00431111* FBP2.

**Fig. 3:**
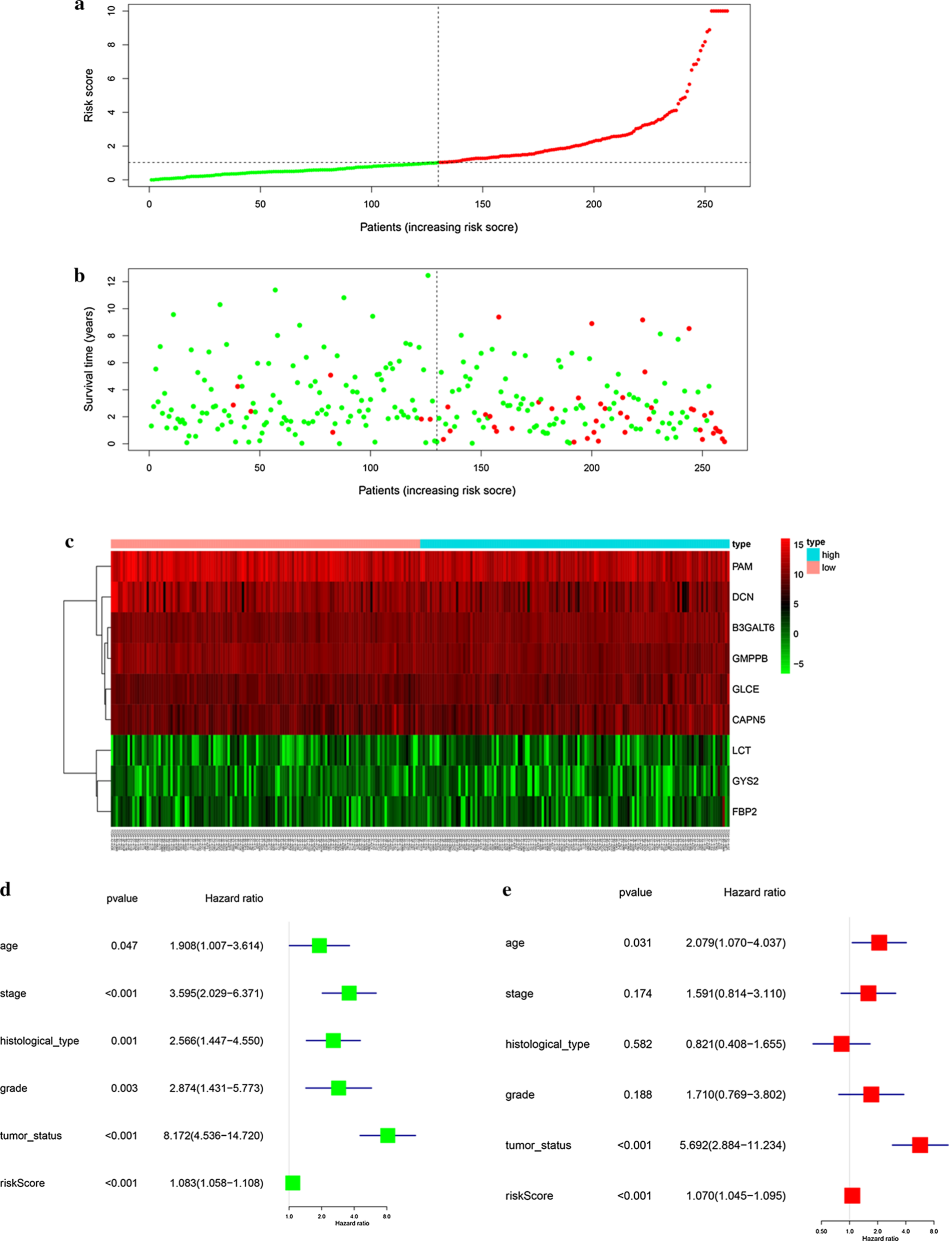
Risk signature with the 9 glycolysis-related hub genes. **a**, **b** The risk scores for all patients in training cohort are plotted in ascending order and marked as low risk (blue) or high risk (red), as divided by the threshold (vertical black line). **c** The distribution of risk score, survival status, and the expression of 9 genes of each patient in training cohort by z-score, with red indicating higher expression and light blue indicating lower expression. **d** Univariate regression model. **e** Multivariate regression model

According to the median levels of risk score, EC patients were classified into low-risk (n = 130) and high-risk groups (n = 130). In the model, survival analysis indicated that low-risk patients had significantly longer overall survival time than high-risk patients (Fig. 2c). We also performed the receiver operating characteristic curve (ROC) analysis. As shown in Fig. 2d, ROC curve analysis was also completed according to the 1, 3, 5-year survival of the area under the receiver operating characteristic curve (AUC) value, the specificity and sensitivity were highest when the risk score was 0.794, 0.765, 0.773. The risk score and survival status indicated by the prognostic model was displayed in Fig. 3a–c. To assess whether the model was an independent predictor of EC, univariate and multivariate Cox regression analyses were conducted, including risk scores and clinical factors. And the results showed that this prognostic model showed moderate and independent prognostic power for Glycolysis pathway (Fig. 3d, e).

### Validation of glycolysis-related genes and EC prognosis

**Fig. 4:**
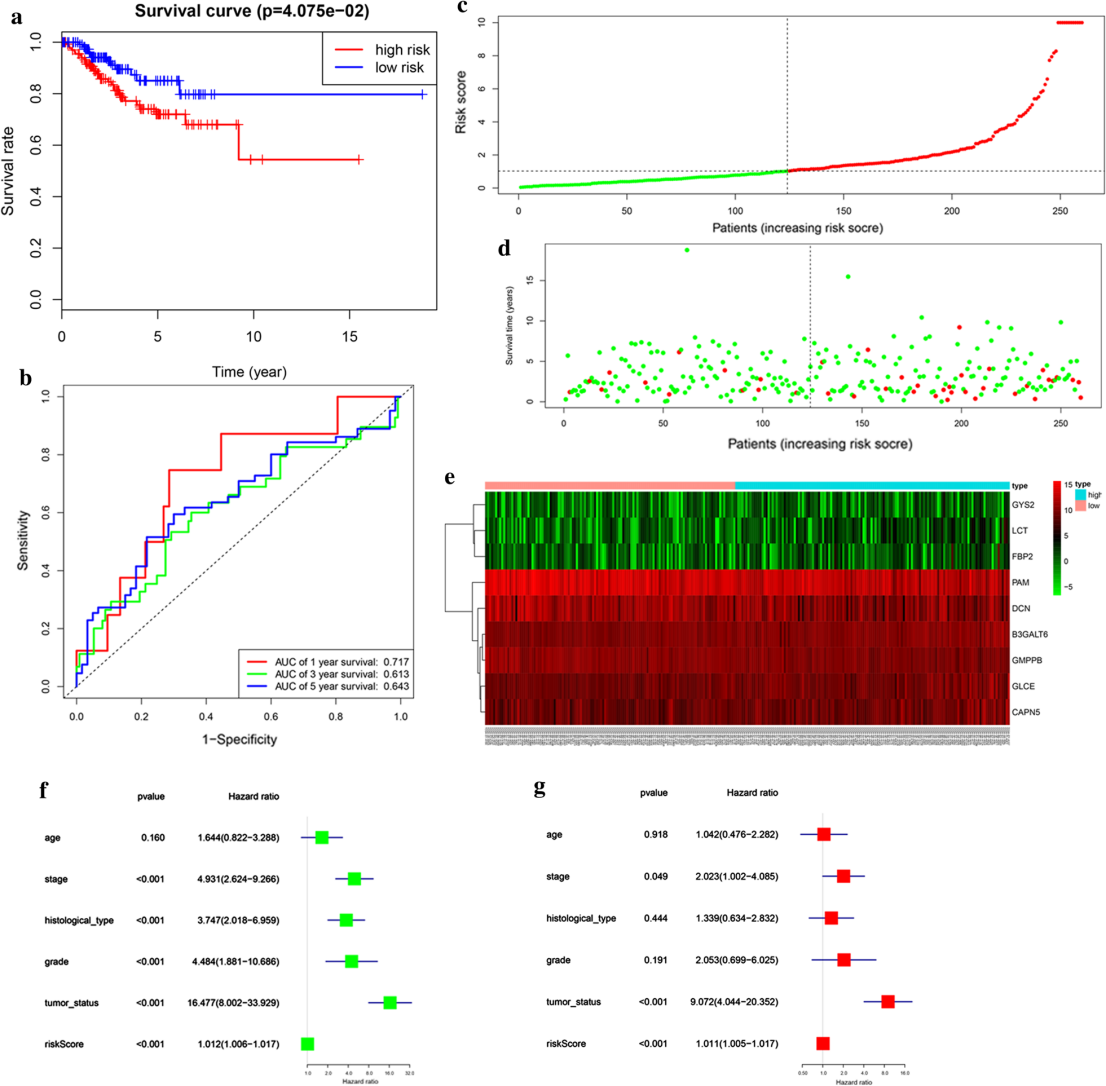
Prognostic model of the testing cohort and risk signature with the 9 glycolysis-related hub genes. **a** Kaplan–Meier survival analysis of the low- and high-risk group patients in the testing cohort. **b** ROC curve analysis according to the 1, 3, 5-year survival of the area under the AUC value. **c**, **d** The risk scores for all patients in testing cohort are plotted in ascending order and marked as low risk (blue) or high risk (red), as divided by the threshold (vertical black line). **e** The distribution of risk score, survival status, and the expression of 9 genes of each patient in testing cohort by z-score, with red indicating higher expression and light blue indicating lower expression. **f** Univariate regression model. **g** Multivariate regression model

In order to verify the authenticity of the above prognostic model, we built another prognostic model using the testing cohort (260 samples). Based on the training cohort’ cut-off, samples were divided into low-risk (n = 124) and high-risk group (n = 136) according to the median levels of risk score. Survival analysis indicated that low-risk patients had significantly longer overall survival time than high-risk patients (Fig. 4a). ROC curve analysis showed that the specificity and sensitivity were highest when the risk score was 0.717, 0.613, 0.643 according to the 1, 3, 5-year survival of the area under the receiver operating characteristic curve (AUC) value (Fig. 4b). The risk score and survival status indicated by the prognostic model was displayed in Fig. 4c–e. To assess whether the model was an independent predictor of EC, univariate and multivariate analyses were completed, including clinical factors and risk scores. The results showed that this prognostic model showed moderate and independent prognostic power for Glycolysis pathway (Fig. 4f, g). These conclusions were all consistent with previous prognostic model trends, validating the reliability of our speculation that Glycolysis is involved in the development of EC and affects the prognosis of EC.

### Complete glycolysis-related prognostic model

**Fig. 5:**
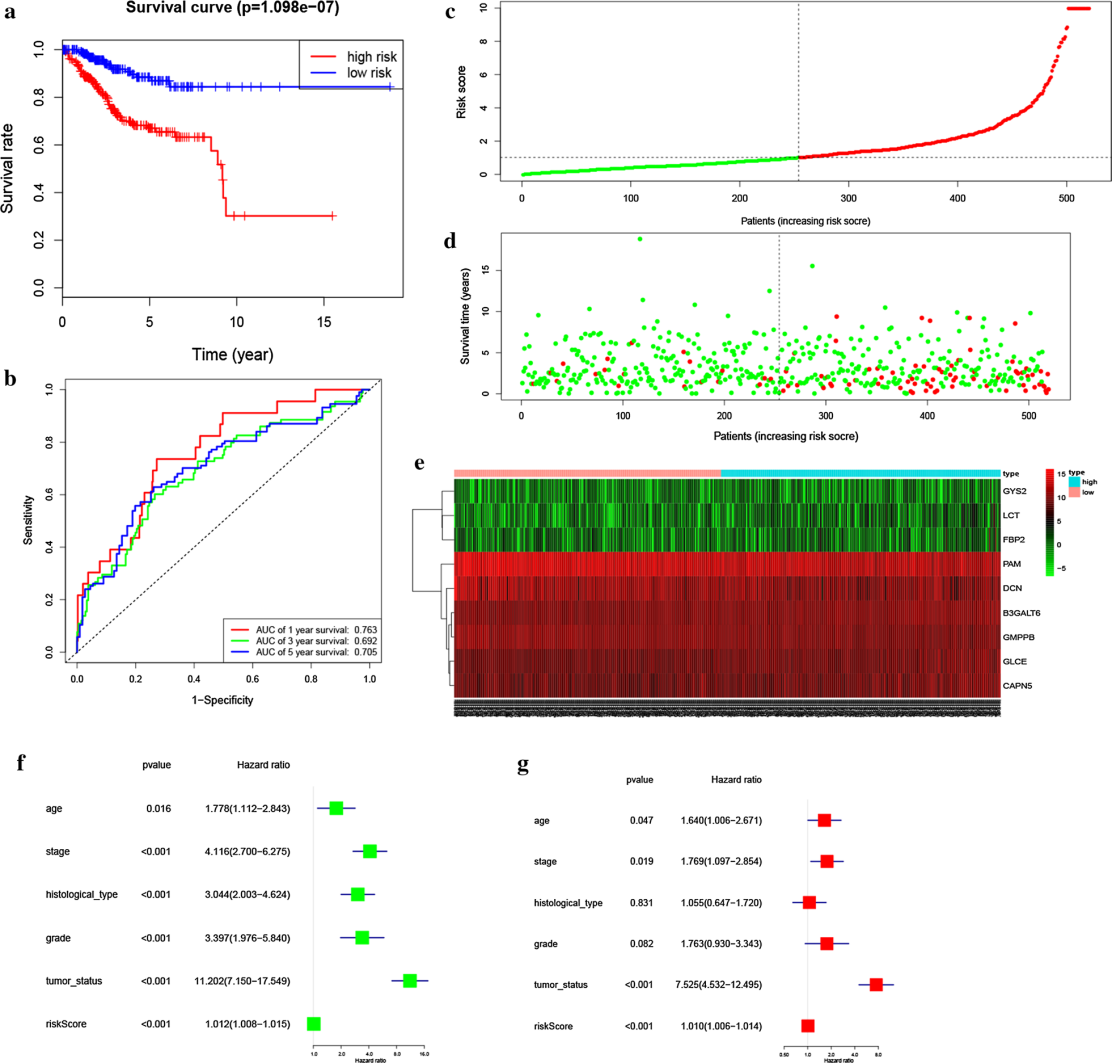
Prognostic model of the entire cohort and Risk signature with the 9 glycolysis- related hub genes. **a** Kaplan–Meier survival analysis of the low- and high-risk group patients in the entire cohort. **b** ROC curve analysis according to the 1, 3, 5-year survival of the area under the AUC value. **c**, **d** The risk scores for all patients in entire cohort are plotted in ascending order and marked as low risk (blue) or high risk (red), as divided by the threshold (vertical black line). **e** The distribution of risk score, survival status, and the expression of 9 genes of each patient in entire cohort by z-score, with red indicating higher expression and light blue indicating lower expression. **f** Univariate regression model. **g** Multivariate regression model

We finally built a complete prognostic model based on entire cohort (520 samples). Based on the training cohort’ cut-off and median levels of risk score, samples were classified into low-risk (n = 254) and high-risk (n = 266) group, and survival analysis indicated that low-risk patients had significantly longer overall survival time than high-risk patients (Fig. 5a). ROC curve analysis showed that the specificity and sensitivity were highest when the risk score was 0.763, 0.692, 0.705 according to the 1, 3, 5-year survival of the area under the receiver operating characteristic curve (AUC) value (Fig. 5b). The risk score and survival status indicated by the prognostic model was displayed in Fig. 5c–e. To assess whether the model was an independent predictor of EC, univariate and multivariate analyses were done, including clinical factors and risk scores. The results showed that this prognostic model showed moderate and independent prognostic power for Glycolysis pathway (Fig. 5f, g). These further validate the reliability of our previous two prognostic models.

### Hierarchical analysis of clinical features and glycolysis-related hub genes

**Fig. 6:**
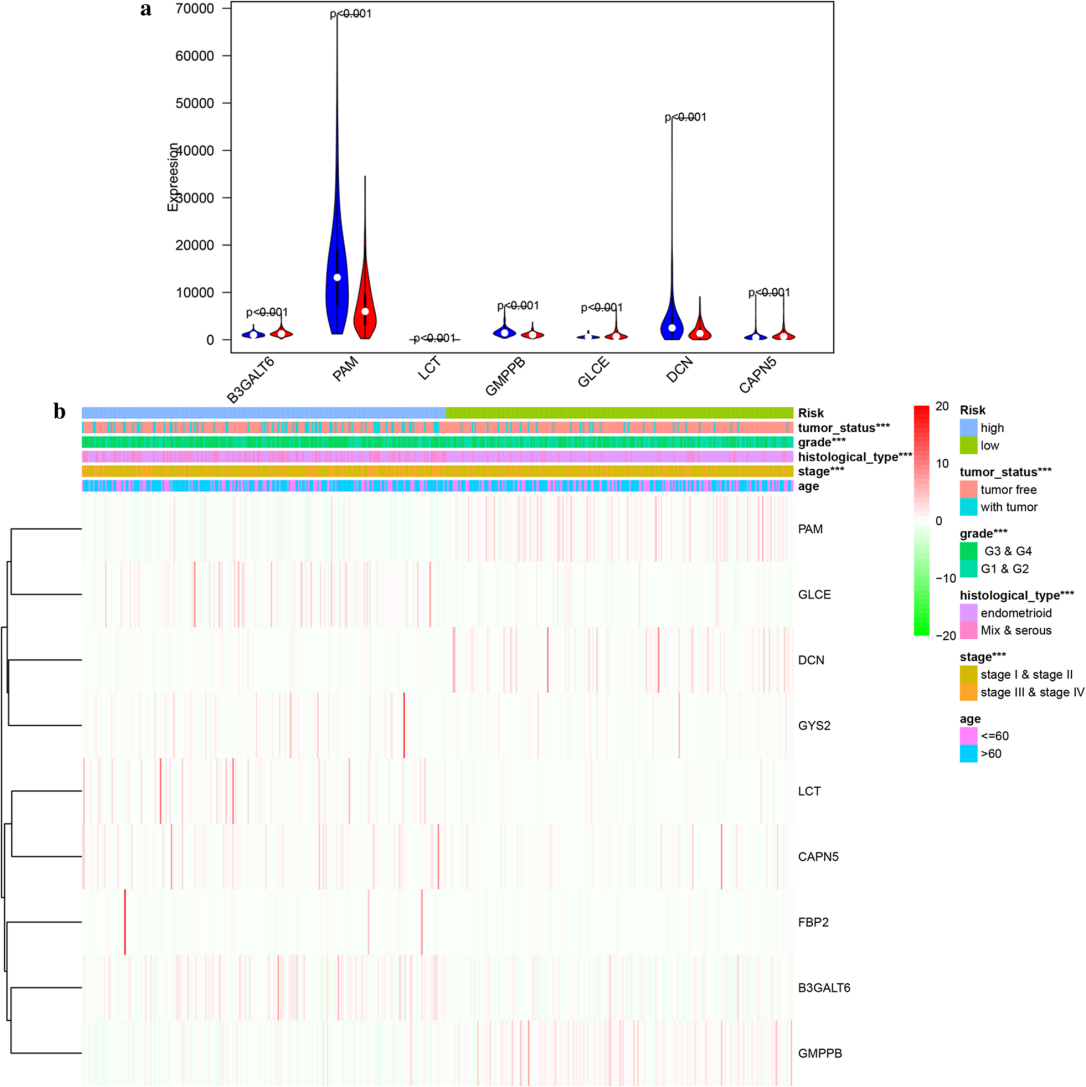
Validation of the 9 glycolysis-related hub genes. **a** Expression level of 7 significant glycolysis-related hub genes in in high- and low-risk groups. **b** Heatmap showed the expression of the 9 glycolysis-related hub genes in high- and low-risk patients in the TCGA dataset associated with tumor status, grade, histological type and stage

**Fig. 7:**
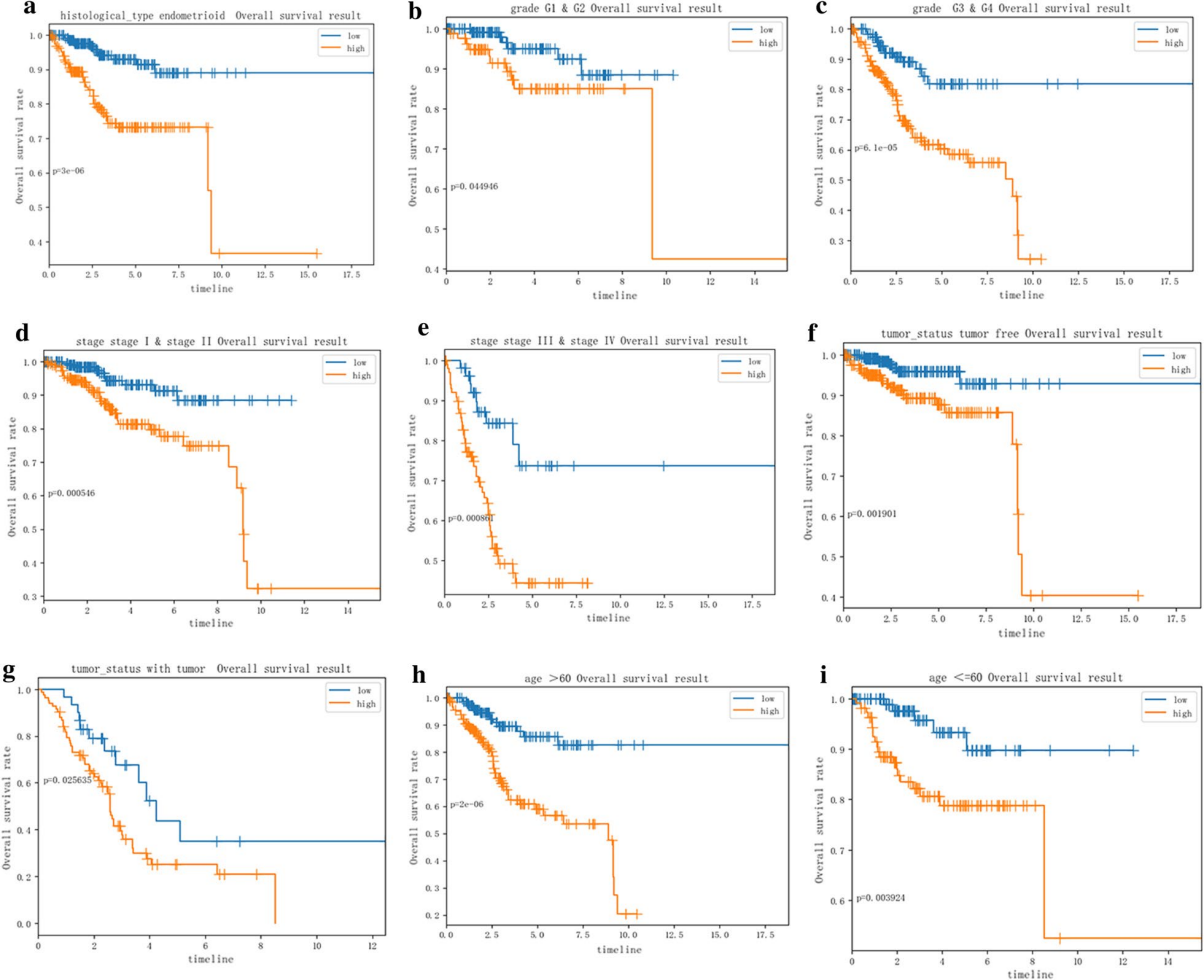
Survival time of patients in high-risk and low-risk group of different subgroups. **a** endometrioid subgroup, **b** grade G1 and G2 subgroup, **c** grade G3 and G4 subgroup, **d** stage I and stage II subgroup, **e** stage III and stage IV subgroup, **f** tumor free subgroup, **g** with tumor subgroup, **h** age > 60 subgroup, (I) age ≤ 60 subgroup

Univariate and multivariate Cox proportional hazards regression analysis identified nine genes including B3GALT6, PAM, LCT, GMPPB, GLCE, DCN, CAPN5, GYS2 and FBP2 to be prognosis-related. Among the 9 genes, we found significant differences in the expression levels of 7 genes in the high-risk and low-risk groups (Fig. 6a). In addition, the heatmap showed the expression of the nine genes in high- and low-risk patients in the TCGA dataset. We observed significant differences between the high- and low-risk groups associated with tumor status, grade, histological type and stage (Fig. 6b). We further analyzed the relationship between nine genes and various clinical features including risk, tumor status, grade, histological type, stage and age. We found that tumor status, grade, histological type and stage were significantly related with the 9 genes. We analyzed 9 genes for different clinical features respectively. We found that expression level of CAPN5, DCN, GLCE and GMPPB were significantly different in different age groups (Additional file 2: Figure S1).For different histological type, the expression level of B3GAL, CAPN5, GLCE, GMPPB and PAM were significantly different (Additional file 3: Figure S2). For different grade, the expression level of CAPN5, DCN, GLCE, GMPPB and PAM were significantly different   (Additional file 4: Figure S3). For different tumor status, DCN, GMPPB and PAM expressed differently  (Additional file 5: Figure S4). In next, the stratification analysis was done according to histological type, grade, stage, tumor status and age. Patients were stratified into endometrioid subgroups, grade G1 and G2 subgroup, grade G3 and G4 subgroup, tumor free subgroup, with tumor subgroup, stage I and stage II subgroup, stage III and stage IV subgroup, age > 60 subgroup and age ≤ 60 subgroup. For the patients in endometrioid subgroup, the survival time of patients in the low-risk case was significantly longer than that of patients in the high-risk case (Fig. 7a), which was consistent with the results belonging to the grade G1 and G2 subgroup, grade G3 and G4 subgroup, stage I and stage II subgroup, stage III and stage IV subgroup, tumor free subgroup, with tumor subgroup, age > 60 subgroup and age ≤ 60 subgroup. (Figure 7b–i).

### Building predictive nomogram

**Fig. 8:**
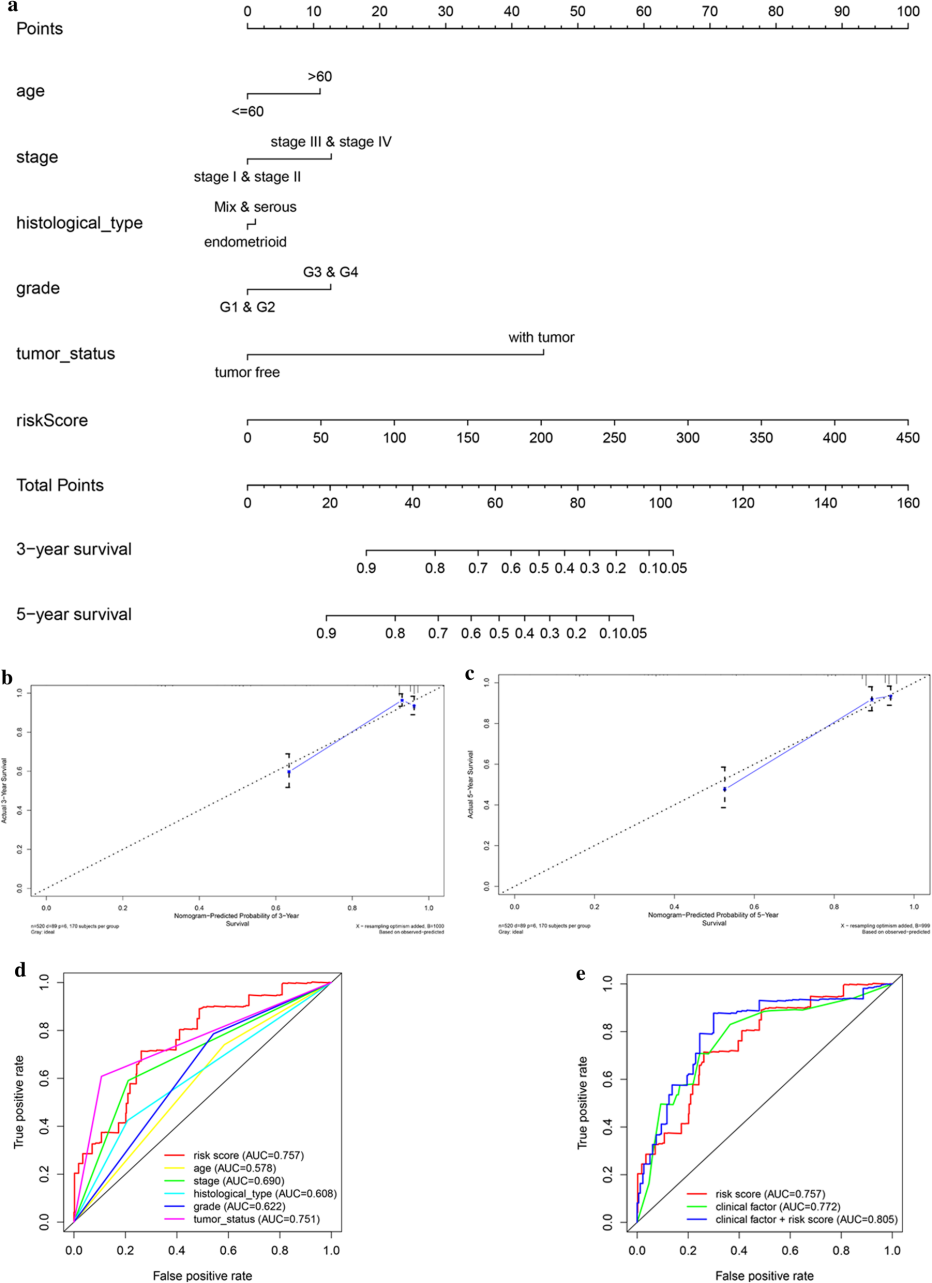
The nomogram to predict 3‐ or 5‐year OS and prognostic value of 9 genes in the entire set. **a** The nomogram for predicting proportion of patients with 3‐ or 5‐year OS. **b**, **c** The calibration plots for predicting patient 3‐ or 5‐year OS. Nomogram‐predicted probability of survival is plotted on the x‐axis; actual survival is plotted on the y‐axis. **d**, **e** The relationship between 9-mRNA signature and different clinical features

For the goal of establishing a clinically method to predict the survival probability with EC patients, we created a nomogram based on the TCGA cohort to estimate the probability of the 3‐ and 5‐year OS. The predictors of the nomogram contained 6 independent prognostic factors including stage, age, histological type, grade, tumor status and risk score Fig. 8a). The C‐index of the model for evaluation of OS was 0.871. The 45° line represented the best prediction. Calibration plots suggested that the nomogram performed well (Fig. 8b–c). ROC curve analysis also showed that the risk score AUC value of the model was 0.757, the clinical factors AUC value was 0.772, both much significantly higher than the clinical stage (AUC = 0.690), grade (AUC = 0.622), histological type (AUC = 0.608), tumor status (AUC = 0.751) and patients’ age (AUC = 0.578). Interestingly, when combined the risk score with clinical factors, the ROC curve of combination model was much higher than each alone (AUC = 0.805).

**Fig. 9:**
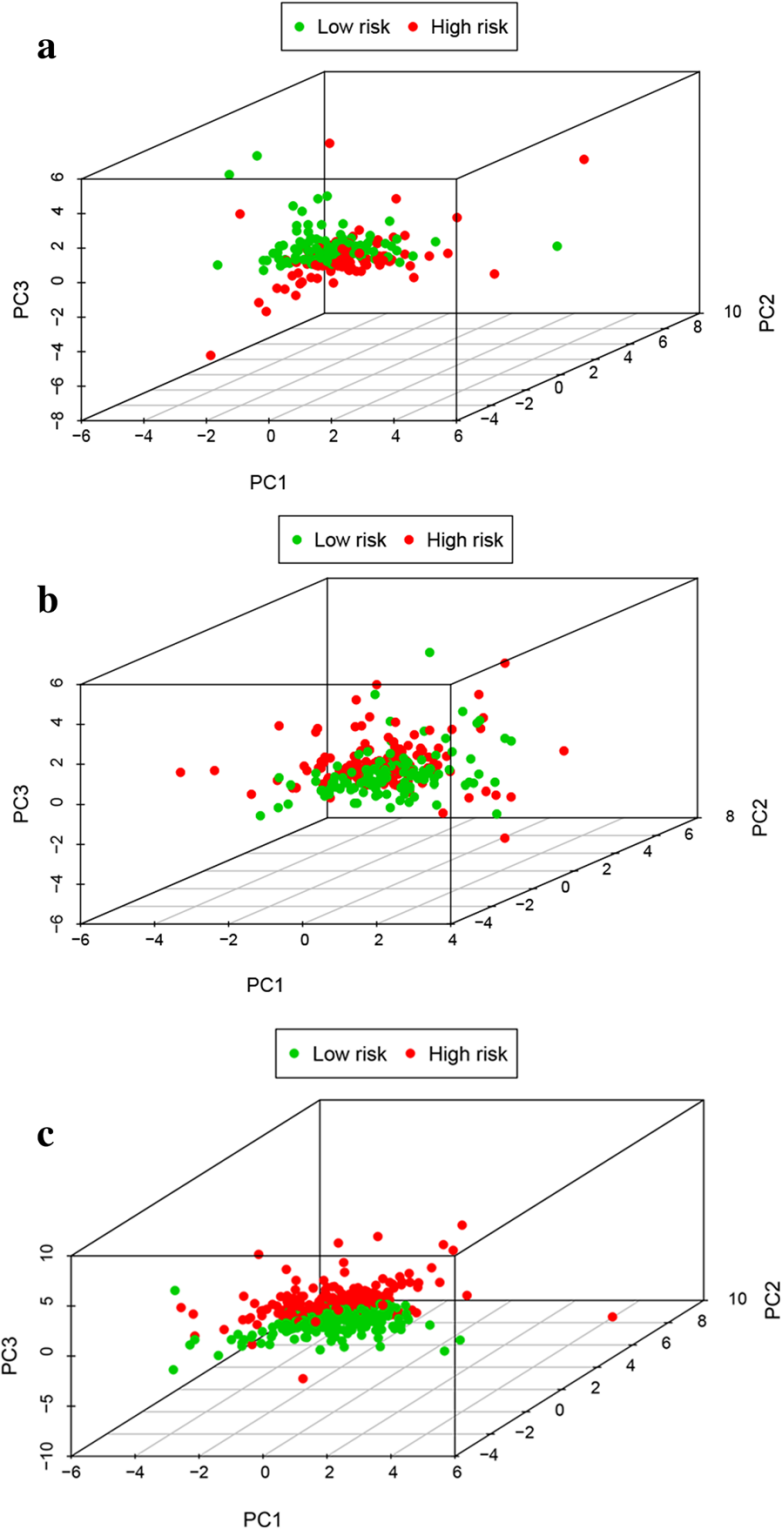
Principal component analysis of the training cohort, testing cohort, and entire EC cohort. **a** Training cohort, **b** testing cohort, **c** entire cohort

Based on 9 glycolysis‐related gene expression, principal component analysis of the training cohort, testing cohort, and entire EC cohort displayed a significantly different distribution pattern of high and low risk which indicating their difference in glycolysis phenotype (Fig. 9a–c).

### Genetic information of the glycolysis-related genes

**Fig. 10:**
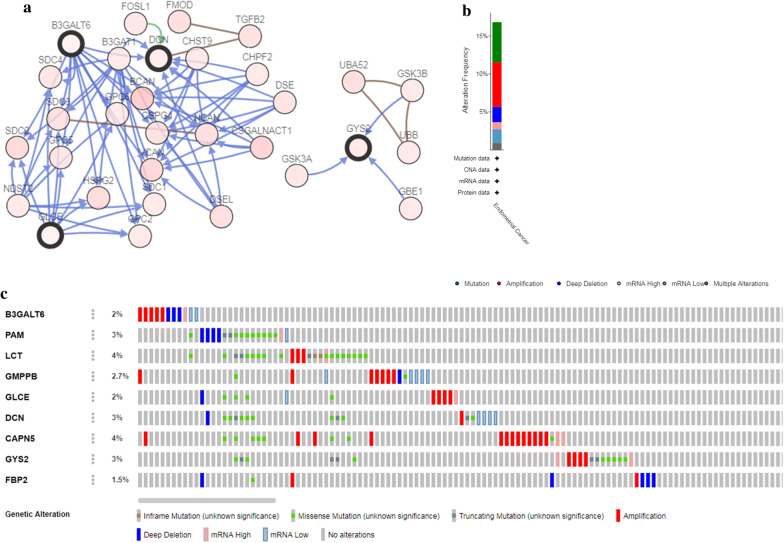
The gene mutation overview of 9 prognostic glycolysis-related genes in TCGA EC patients. **a** The network of 9 genes and the most frequently altered neighbor genes. **b** The summary of mutation type of 9 genes in EC patients. **c** Nine genes were altered in 92 (17%) from the 547 patients

The genetic alteration in the Glycolysis-related genes was analyzed with cBioPortal software. The network constructed by B3GAL, DCN, GLCE, GYS2 and their most associated neighbor genes were exhibited (only four out of the 9 genes had a joint node, while the remaining 5 genes had no junctions and were not shown) (Fig. 10a). Figure 10b, c illustrated that the 9 genes were altered in 92 (17%) from the 547 patients; LCT and CAPN5 showed most diverse alteration including amplification, missense mutation etc.

### Validation of glycolysis-related hub genes

**Fig. 11:**
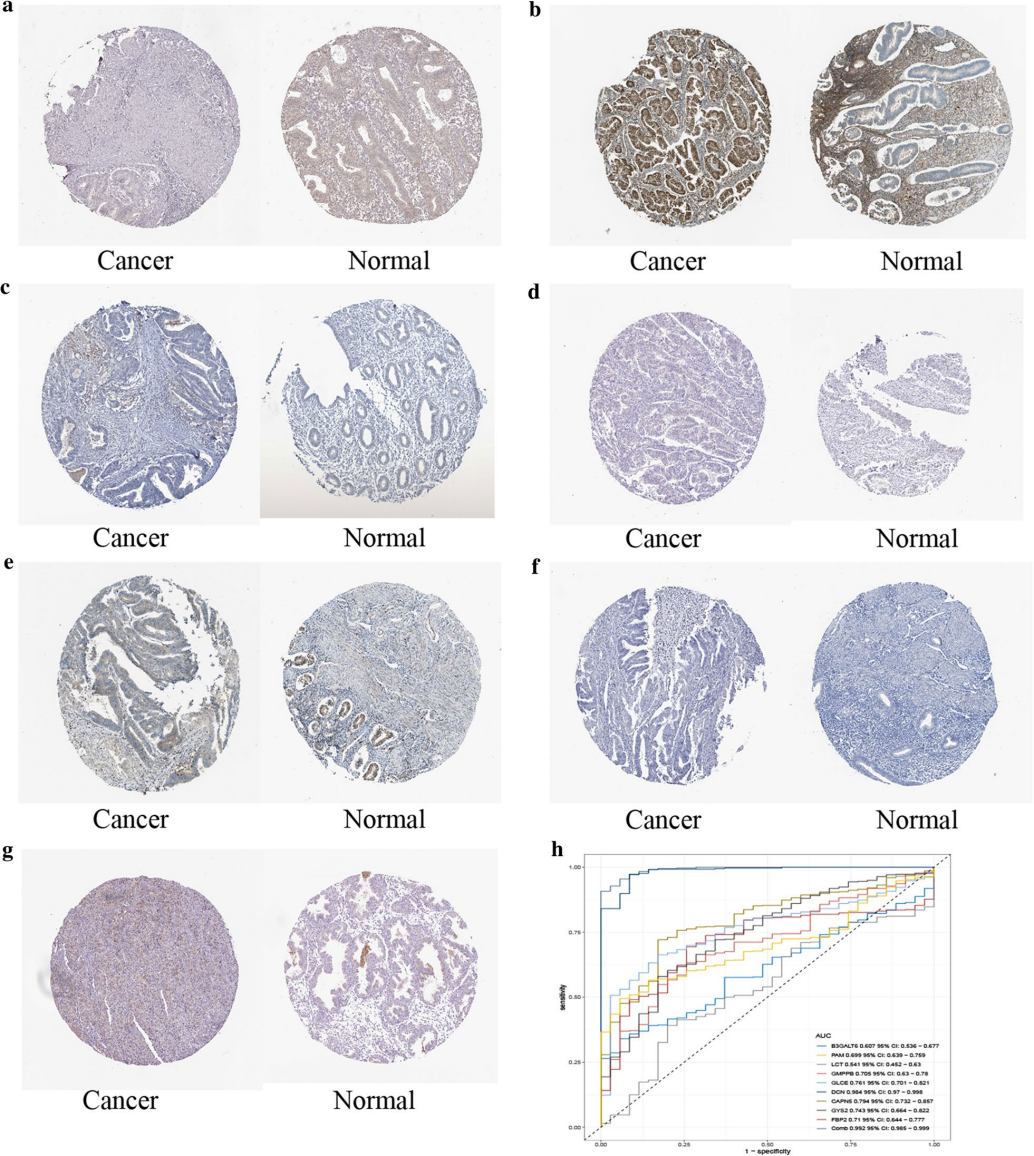
The protein expression difference of **a** CAPN5, **b** DCN, **c** FBP2, **d** GLCE, **e** GMPPB, **f** GYS2, **g** PAM between EC and normal samples from TCGA. **h** AUC value was used to identify the diagnostic efficacy of distinguishing normal and cancerous tissues

**Fig. 12:**
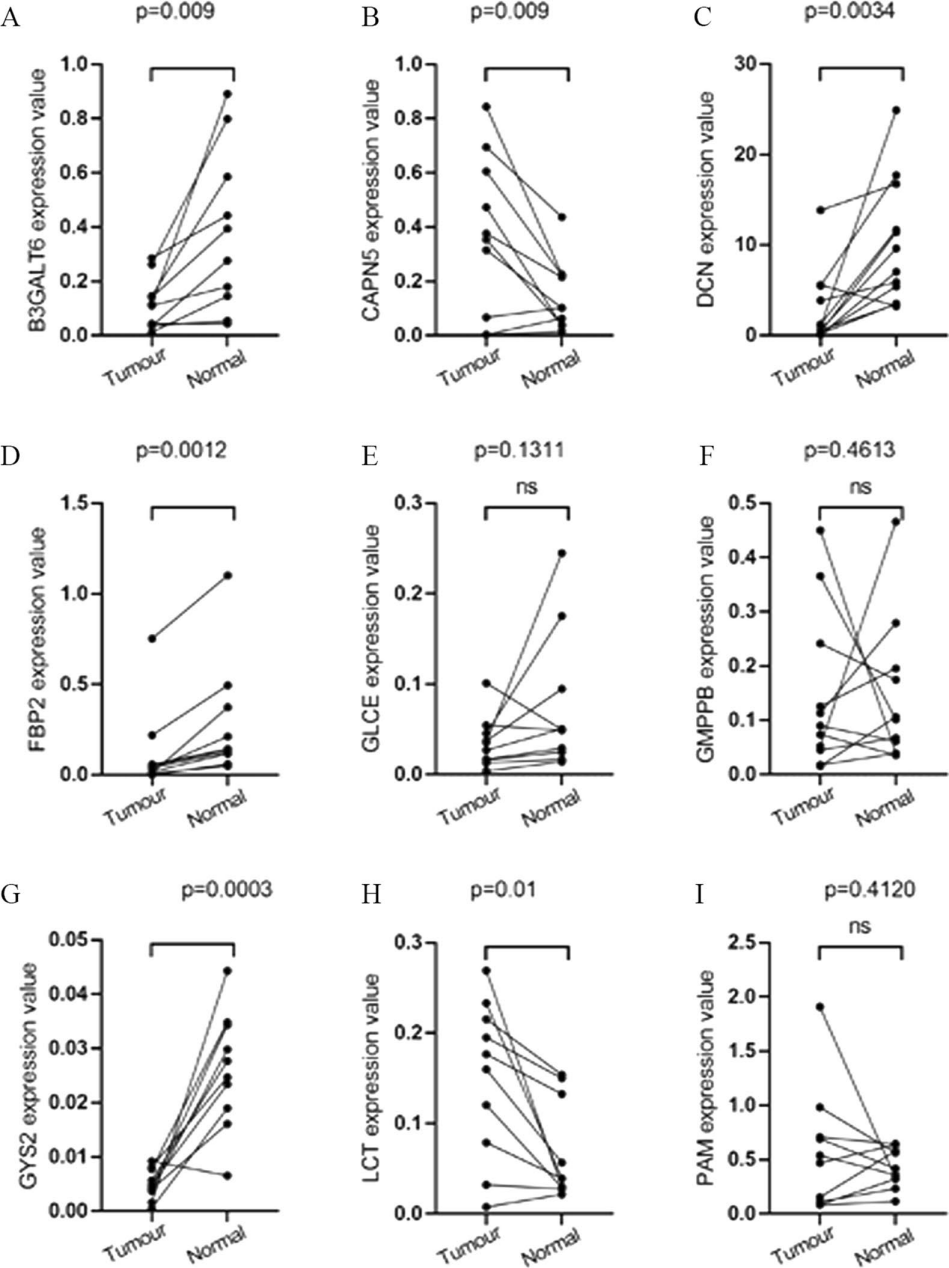
Verification of expression levels of 9 glycolysis-related genes in tumor and normal tissue samples. **a** B3GALT6, **b** CAPN5, **c** DCN, **d** FBP2, **e** GLCE, **f** GMPPB, **g** GYS2, **h** LCT, **i** PAM

All of the 9 glycolysis-related hub genes were validated in TCGA data. We found that DCN had the lower expression level of EC tissues than that of healthy tissues, while the B3GALT6, PAM, LCT, GMPPB, GLCE, CAPN5, GYS2 and FBP2 had the higher expression levels of EC tissues than that of healthy tissues (Additional file 6: Figure S5). We further validated the 9 glycolysis-related hub genes including B3GALT6, PAM, LCT, GMPPB, GLCE, DCN, CAPN5, GYS2 and FBP2 using immunohistochemistry. PAM, GMPPB, GLCE, CAPN5, GYS2 and FBP2 had the consistent expression trend. B3GALT6 and LCT were not available in the database (Fig. 11a–g). AUC value was used to identify the diagnostic efficacy of distinguishing normal and cancerous tissues, AUC value of 9 genes combined diagnosis was 0.992, which means the 9 genes can well identify cancer tissue and normal tissue (Fig. 11h). Regarding prognosis, Kaplan–Meier curves showed that higher expression of CAPN5, FBP2 and GYS2 correlated significantly with poor overall survival (OS), while the lower expression of DCN, GMPPB and PAM correlated significantly with OS (Additional file 7: Figure S6). We further verified the expression of these 9 genes in clinical sample tissues (Fig. 12). The results prove that the expression levels of B3GALT6, DCN, FBP2 and GYS2 are lower in tumor samples and higher in normal tissue samples. The expression of CAPN5 and LCT in clinical sample tissues is just the opposite.

### Association between nine hub genes and genetic mutations

We compared the frequency of genetic mutations between high- and low-risk score groups through R package maftools. The high-risk group had somatic mutations in the following order: TP53 > PTEN > PIK3CA > ARID1A > TTN > PIK3R1 > KMT2D > CTNNB1 > CTCF > MUC16 (Additional file 6: Figure S5). The low-risk group had somatic mutations in the following order: PTEN > ARID1A > PIK3CA > TTN > PIK3R1 > CTCF > KMT2D > ZFHX3 > MUC16 > MUC5B (Additional file 6: Figure S5). Furthermore, we found that the patients in the low-risk group showed obvious mutation signatures, compared with patients in the high-risk group (Additional file 8: Figure S7).

### Identification of nine cell cycle-related genes risk score associated biological pathways

**Fig. 13:**
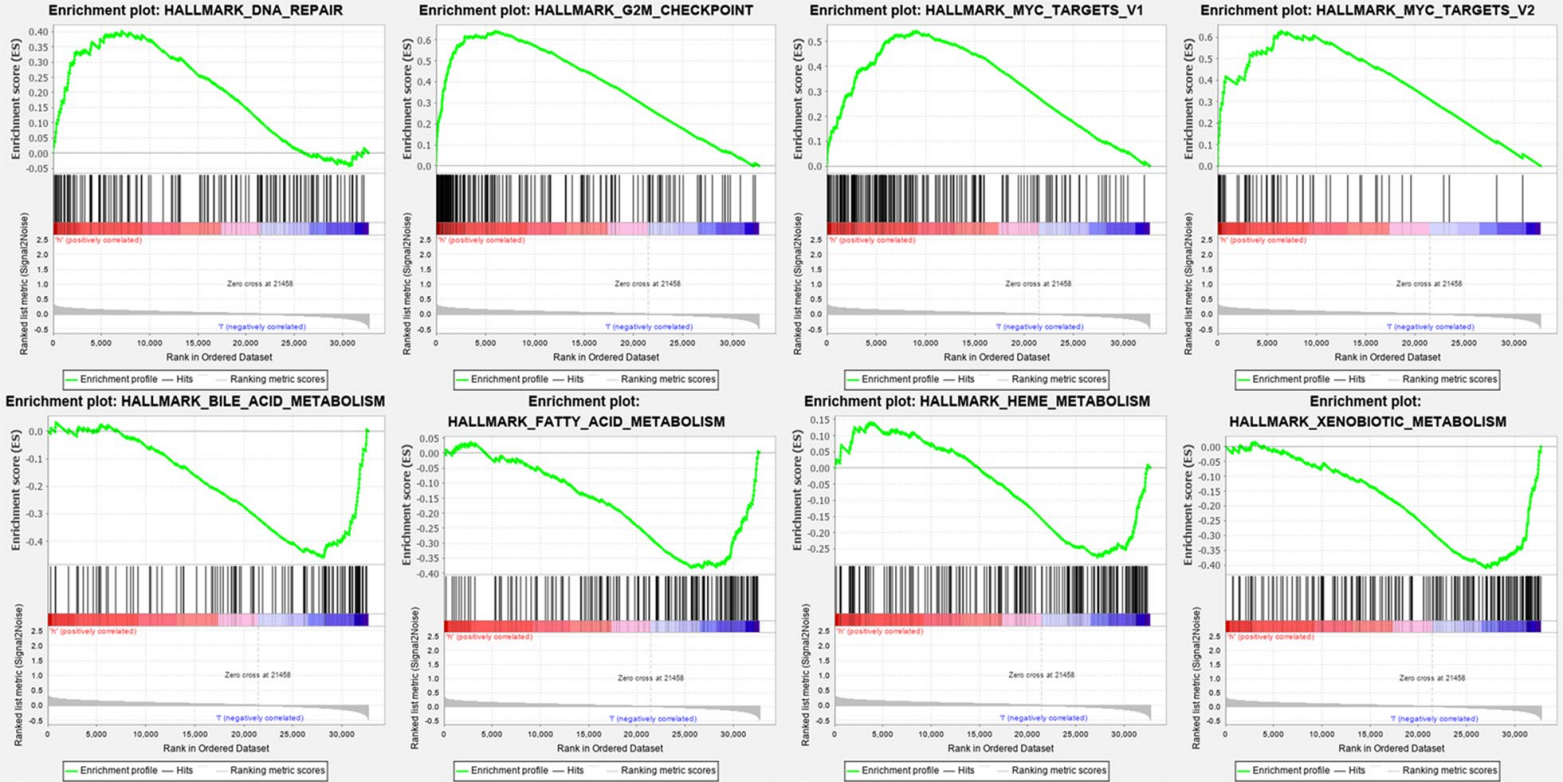
Gene Set Enrichment Analysis in TCGA database. Enrichment Map were used for visualization of the GSEA results

GSEA further analyzes high- and low-risk group samples, revealing the main enrichment pathway. The samples of the high-risk group were mainly concentrated on the pathways such as dna repair, g2m checkpoint, myc targets v1 and myc targets v2. The samples of the low-risk group were mainly concentrated on the pathways such as bile acid metabolism, fatty acid metabolism, heme metabolism and xenobiotic metabolism (Fig. 13).

## Discussion

Endometrial cancer is a common malignant tumor that threatens women’s lives. It often occurs in postmenopausal women and is difficult to diagnose in the early stage. Therefore, it needs to be widely regarded. Glycolysis is a common energy metabolism pathway in human body. Many studies have shown that Glycolysis affected the biological behavior of tumor cells. We reasonably speculated that Glycolysis was related to the development of endometrial cancer, so we analyzed endometrial cancer samples by GESA. Glycolysis was found to be the most highly enriched pathway, initially confirming our hypothesis.

EC samples were randomly classified into training cohort, testing cohort and entire cohort. We used training cohort to construct Cox regression prognostic model, testing cohort and entire cohort for validation. Nine glycolysis-related prognostic genes including B3GALT6, PAM, LCT, GMPPB, GLCE, DCN, CAPN5, GYS2 and FBP2 were screened out. After a comprehensive analysis of the clinical information, we found that these nine genes are associated with multiple clinical features of EC respectively.

After reviewing the existing literature, we found that these nine genes are more or less related to tumors. For example, Saldise et al. found that the distribution of peptidyl-glycine alpha-amidating mono-oxygenase (PAM) enzymes in normal human lung and in lung epithelial tumors were different [18]. Lactase-phlorizin hydrolase was researched to be associated with colorectal cancer patients by Piepoli A [19]. d-glucuronyl C5-epimerase (GLCE) was shown as a potential tumor suppressor gene which participated in lung and breast carcinogenesis [20, 21] by inhibiting tumor angiogenesis and invasion/metastasis pathways, which was also proved to affect angiogenesis in prostate cancer cells [22]. Decorin (DCN), as an important component of the extracellular matrix (ECM), is a small leucine-rich proteoglycan and synthesized by fibroblasts, the deficiency of which promoted renal cell carcinoma growth and metastasis [23]. DCN was also seen to be potential biomarker of Colon Cancer [24]. Zhang et al.’s research proves that DCN affects the microenvironment of tumors [25]. glycogen synthase 2 (GYS2) was demonstrated to participate in a feedback loop which restricted HBV-Related Hepatocellular Carcinoma growth [26]. Far-upstream element (FUSE)-binding protein 2 (FBP2) belongs to single-stranded DNA-binding protein family; it usually acts in regulating transcription and post-transcription and has been widely learned in liver tumors [27, 28] Kajiwara et al. demonstrated that in colon cancer tissues c-myc suppressor far-upstream element-binding protein-interacting repressor splicing variants were activated [29], which was also proved to induce invasion and migration of non-small cell lung cancer cells [30]. Wang et al. found that FBP2 was correlated with proliferation and doxorubicin resistance in human breast cancer cell lines [31]. B3GALT6, GMPPB and CAPN5 have not been thoroughly studied in tumors.

The somatic mutations analysis between samples of high- and low-risk group were conducted, the result of which showed that the different mutated genes could contribute to the different sore of genes in the EC patients. The mutation rate of PTEN, ARID1A and PIK3R1 in the low-risk group is higher than that in thehigh-risk group. Interestingly, these three genes have been proved to have a certain tumor suppressive effect by previous studies [32–34]. PIK3CA has been found to play a role in gynecological tumors such as cervical cancer [35]. The mutations of other genes have not been explored in EC, and it is worth studying in detail in the future.

GSEA displayed that the samples of the high-risk group were mainly concentrated on the pathways such as dna repair, while the samples of the low-risk group were mainly concentrated on the pathways such as bile acid metabolism. Research proved that when DNA repair fails, this damage can lead to carcinogenesis and tumor genomic instability. In this pathway, biological targets involved in immunotherapy can be found [36]. Bile Acid Metabolism has been found to be related to Signaling in Cholestasis, Inflammation, and Cancer [37].

The advantage of this study is that, firstly, it was found that the Glycolysis pathway is related to the mechanism of EC, opening a new perspective for the regulation of metabolic processes and the treatment of EC. Secondly, we found hub genes closely related to EC survival in this pathway. Most of these genes have been confirmed to affect tumor progression and are likely to be used for targeted therapy. B3GALT6, GMPPB and CAPN5 have not been thoroughly studied. We firstly discovered that these three genes were related to EC and might become an innovative research direction in the future.

## Conclusion

This study found that the Glycolysis pathway is associated with EC and screened for hub genes on the Glycolysis pathway, which may serve as new target for the treatment of EC.

## Supplementary information

**Additional file 1: Table S1.** Primer sequence of genes in qRT-PCR.

**Additional file 2: Figure S1.** Expression level of CAPN5, DCN, GLCE and GMPPB in different age groups. (A) CAPN5, (B) DCN, (C) GLCE, (D) GMPPB.

**Additional file 3: Figure S2.** Expression level of B3GAL, CAPN5, GLCE, GMPPB and PAM in different histological type. (A) B3GAL, (B) CAPN5, (C) GLCE, (D) GMPPB, (E) PAM.

**Additional file 4: Figure S3.** Expression level of CAPN5, DCN, GLCE, GMPPB and PAM in different grade. (A) CAPN5, (B) DCN, (C) GLCE, (D) GMPPB, (E) PAM.

**Additional file 5: Figure S4.** Expression level of DCN, GMPPB and PAM in different tumor status. (A) DCN, (B) GMPPB, (C) PAM.

**Additional file 6: Figure S5.** TCGA Expression level Validation of 9 glycolysis-related hub genes. (A) B3GALT6, (B) DCN, (C) FBP2, (D) GLCE, (E) GMPPB, (F) GYS2, (G) LCT, (H) PAM. (I) CAPN5.

**Additional file 7: Figure S6.** Kaplan–Meier curves showed that higher expression of CAPN5, FBP2 and GYS2 correlated significantly with poor OS, while the lower expression of DCN, GMPPB and PAM correlated significantly with OS. The yellow line indicates samples with highly expressed genes (above best-separation value), and the green line designates the samples with lowly expressed genes (below best-separation value).

**Additional file 8: Figure S7.** Somatic mutation analysis. (A) Oncoplot displaying the somatic landscape of EC with high-risk score. (B) Oncoplot displaying the somatic landscape of EC with low-risk score. Stacked bar chart and cohort summary plot displaying distribution of variants according to variant classification, type, and SNV class. Bottompart (from left to right) indicates mutation load for each sample, variant classification type of the high-risk group (C) and low-risk group (D).

## Data Availability

The data and materials can be found from the first author and corresponding author.

## Abbreviations

EC: Endometrial cancer
OS: Overall survival
ROC: Receiver operating characteristic
HRs: Hazard ratios
GSEA: Gene set enrichment analysis
