# Supplementary material for: Nine glycolysis-related gene signature predicting the survival of patients with endometrial adenocarcinoma
Source: Cancer Cell Int. 2020 May 24;20:183. doi: 10.1186/s12935-020-01264-1 (PMC7247270; doi:10.1186/s12935-020-01264-1)
Supplement: Supplementary file 1 — Additional file 1: Table S1. Primer sequence of genes in qRT-PCR. [file 12935_2020_1264_MOESM1_ESM.docx]

**Table S1 Primer sequence of genes in qRT-PCR**

|  | Forward sequence | Reverse sequence |
| --- | --- | --- |
| B3GALT6 | GACGCCTACGAAAACCTCAC | CCTTGAGCACGAACTCGAAG |
| PAM | CTGGGGTTACACCTAAACAGTC | GCTTGAAGTCAATCACGAAGGC |
| LCT | ATCCAGACGAGAAAACAGTGC | GTCAGCAAAGGCTTCGGTTC |
| GMPPB | GGGAATCCGAATCTCCATGTC | GTCTCAGAGAGTAGGTCACGG |
| GLCE | GCAGCTCGGGTCAACTATAAG | GAACGCCGTGGAAACTGGA |
| DCN | ATGAAGGCCACTATCATCCTCC | GTCGCGGTCATCAGGAACTT |
| CAPN5 | CGCCACTGACGACTCACTC | CTGCCACAAACCAGCAGTTG |
| GYS2 | TGAAGTTGCTTGGGAAGTGAC | AGGTTCACACTGTTCCACCTG |
| FBP2 | ACCCGCTACGTTATGGAAAAG | GCCGTCAGCATTGAGTTCAG |
