# Supplementary figures and images for: Nine glycolysis-related gene signature predicting the survival of patients with endometrial adenocarcinoma
Source: Cancer Cell Int. 2020 May 24;20:183. doi: 10.1186/s12935-020-01264-1 (PMC7247270; doi:10.1186/s12935-020-01264-1)

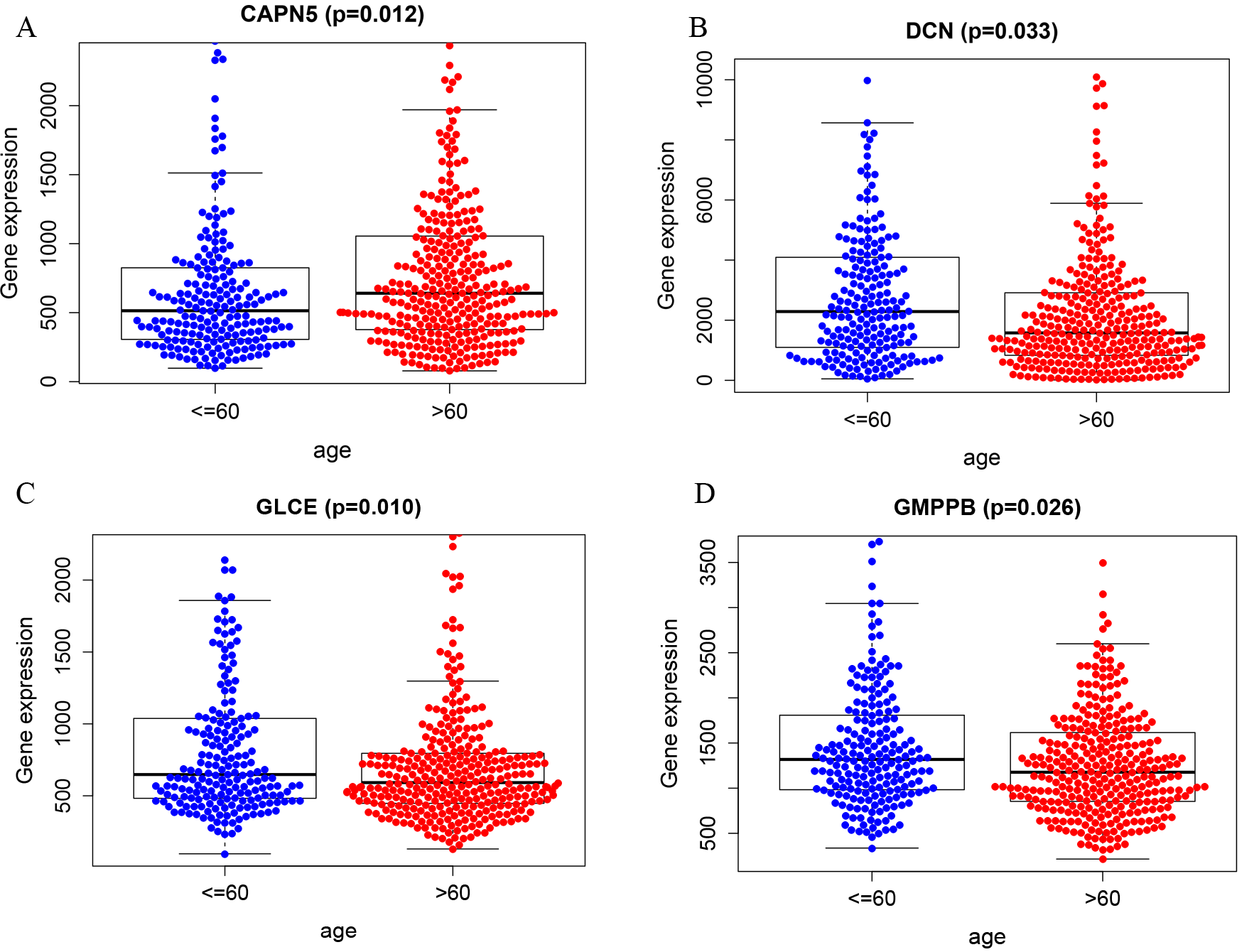

Supplement: Supplementary file 2 — Additional file 2: Figure S1. Expression level of CAPN5, DCN, GLCE and GMPPB in different age groups. (A) CAPN5, (B) DCN, (C) GLCE, (D) GMPPB. [file 12935_2020_1264_MOESM2_ESM.tif]

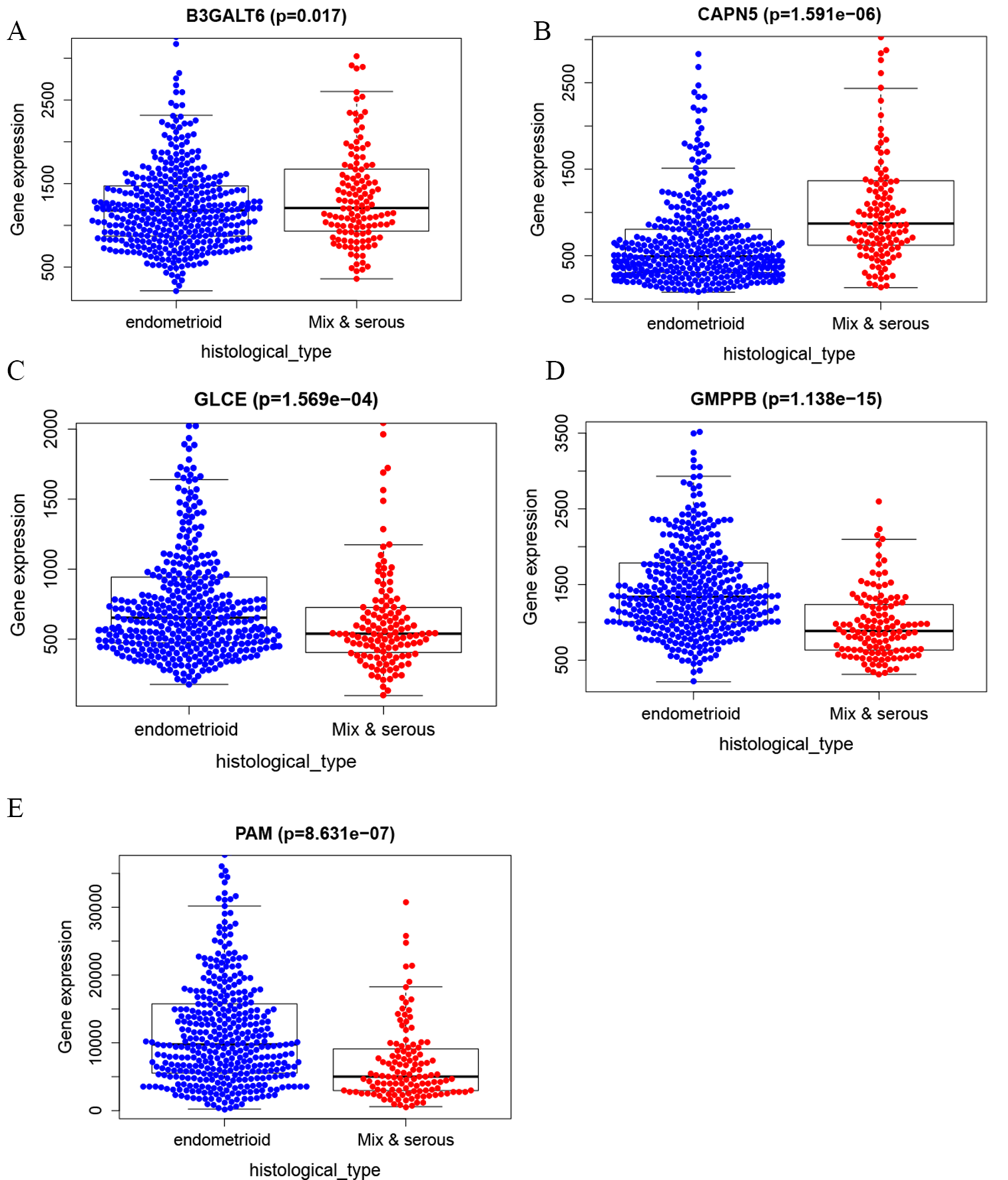

Supplement: Supplementary file 3 — Additional file 3: Figure S2. Expression level of B3GAL, CAPN5, GLCE, GMPPB and PAM in different histological type. (A) B3GAL, (B) CAPN5, (C) GLCE, (D) GMPPB, (E) PAM. [file 12935_2020_1264_MOESM3_ESM.tif]

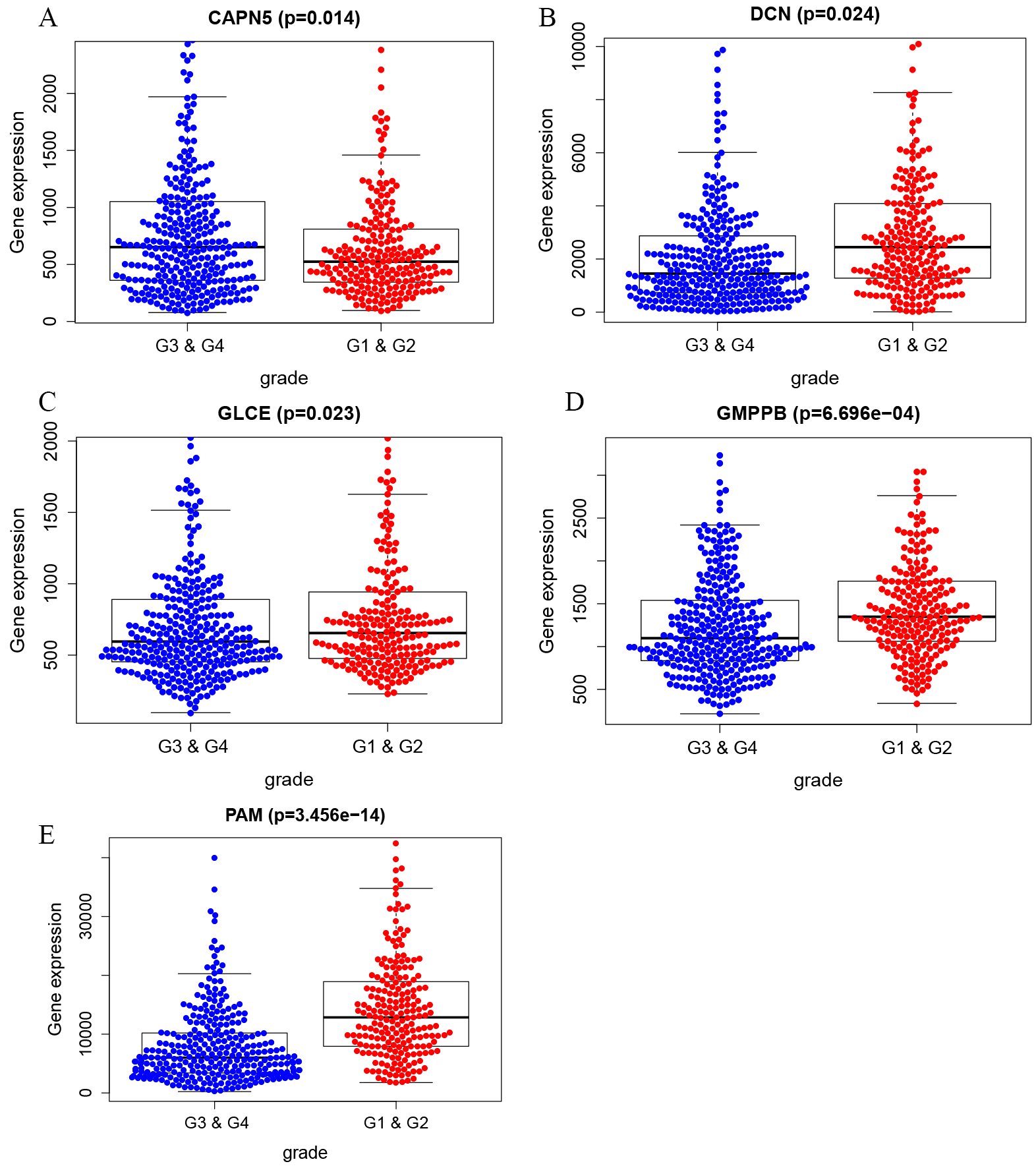

Supplement: Supplementary file 4 — Additional file 4: Figure S3. Expression level of CAPN5, DCN, GLCE, GMPPB and PAM in different grade. (A) CAPN5, (B) DCN, (C) GLCE, (D) GMPPB, (E) PAM. [file 12935_2020_1264_MOESM4_ESM.tif]

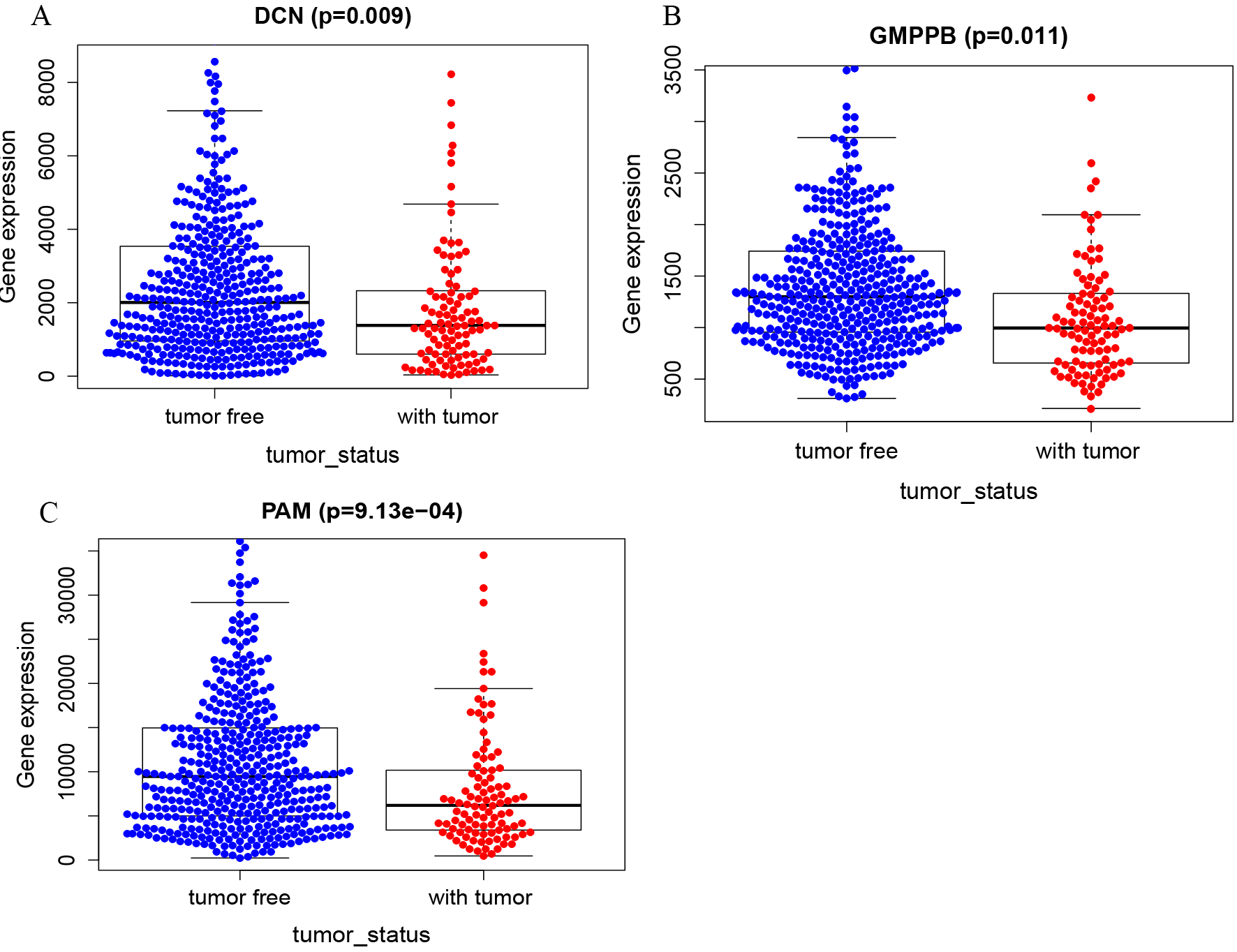

Supplement: Supplementary file 5 — Additional file 5: Figure S4. Expression level of DCN, GMPPB and PAM in different tumor status. (A) DCN, (B) GMPPB, (C) PAM. [file 12935_2020_1264_MOESM5_ESM.tif]

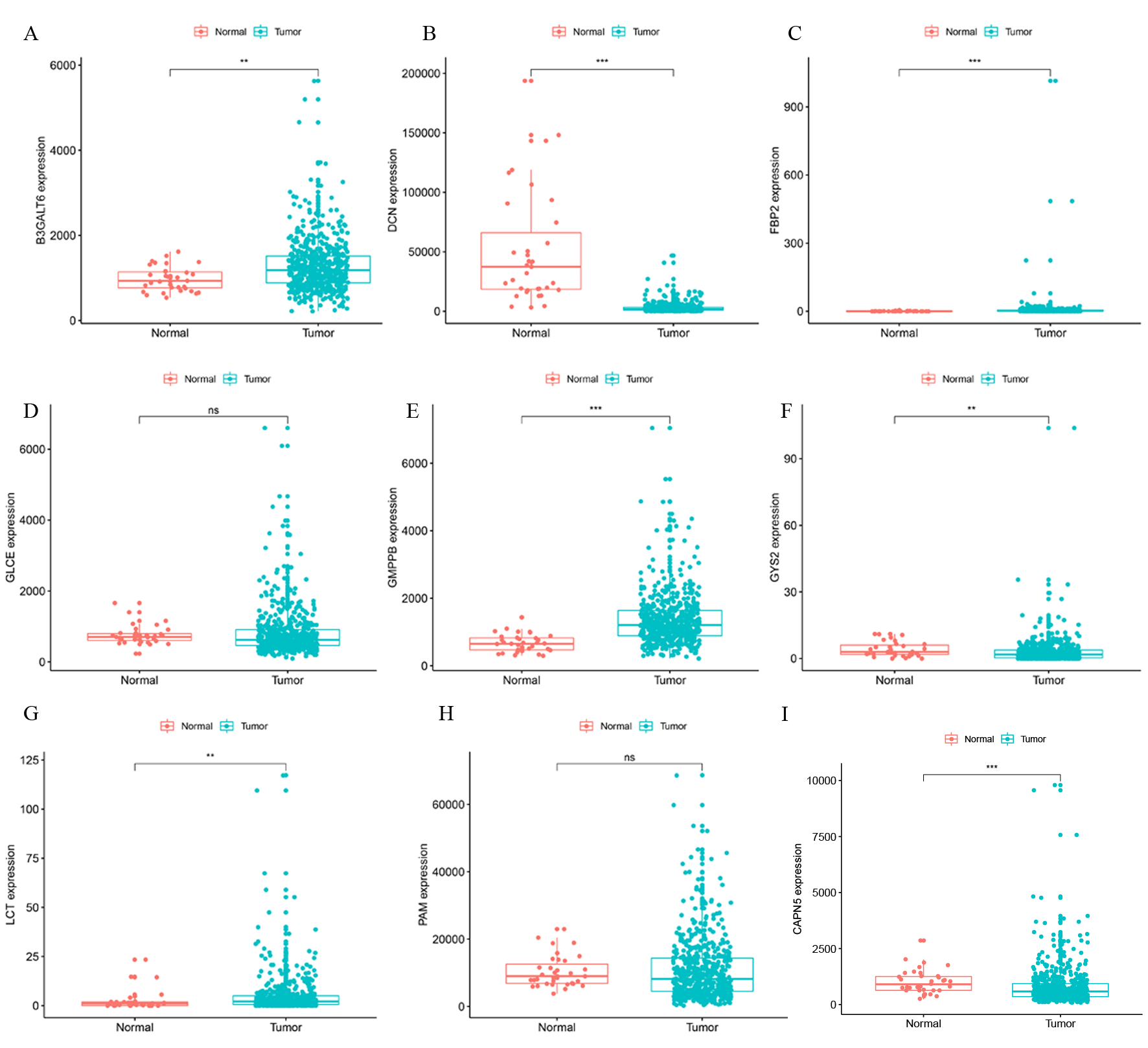

Supplement: Supplementary file 6 — Additional file 6: Figure S5. TCGA Expression level Validation of 9 glycolysis-related hub genes. (A) B3GALT6, (B) DCN, (C) FBP2, (D) GLCE, (E) GMPPB, (F) GYS2, (G) LCT, (H) PAM. (I) CAPN5. [file 12935_2020_1264_MOESM6_ESM.tif]

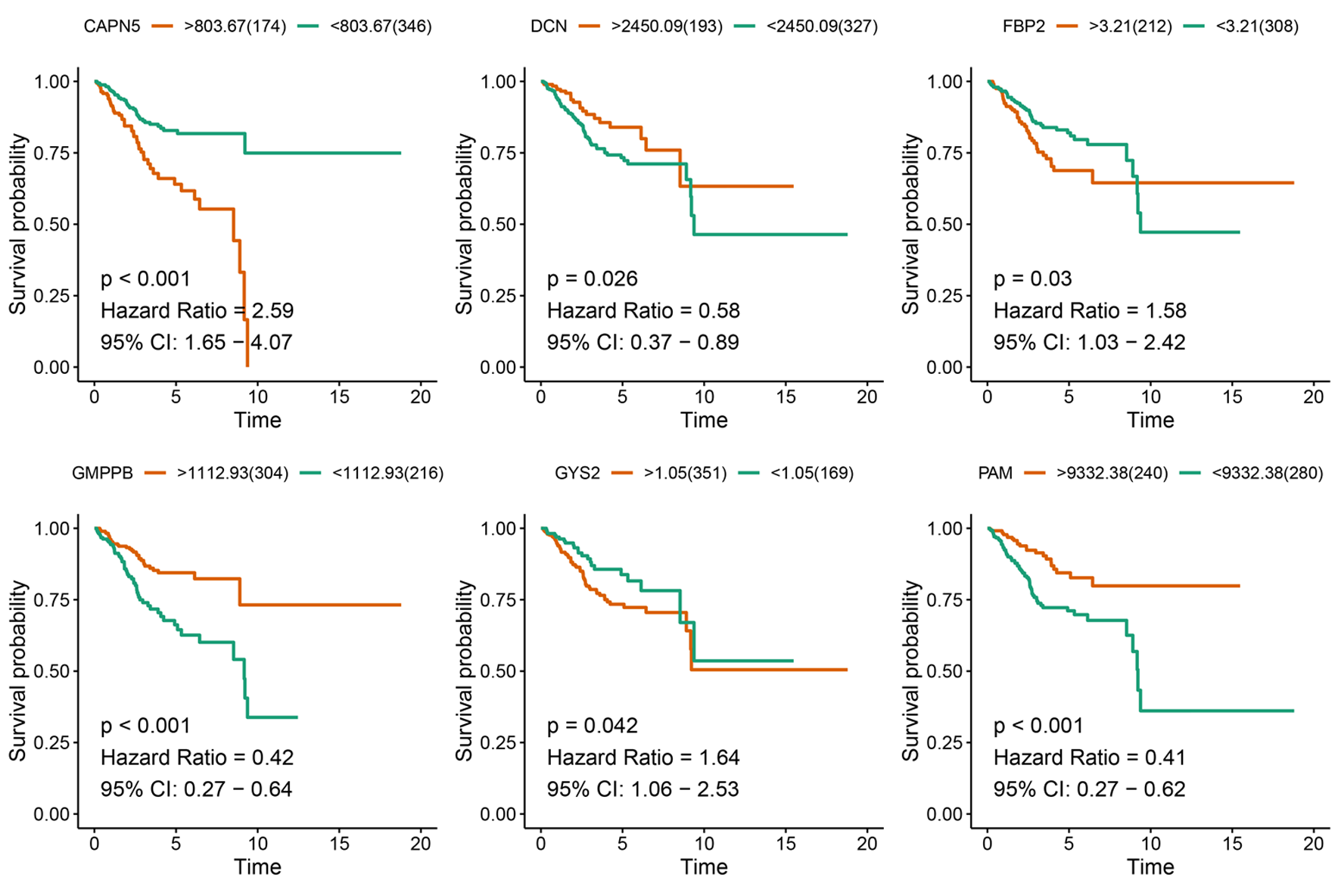

Supplement: Supplementary file 7 — Additional file 7: Figure S6. Kaplan–Meier curves showed that higher expression of CAPN5, FBP2 and GYS2 correlated significantly with poor OS, while the lower expression of DCN, GMPPB and PAM correlated significantly with OS. The yellow line indicates samples with highly expressed genes (above best-separation value), and the green line designates the samples with lowly expressed genes (below best-separation value). [file 12935_2020_1264_MOESM7_ESM.tif]

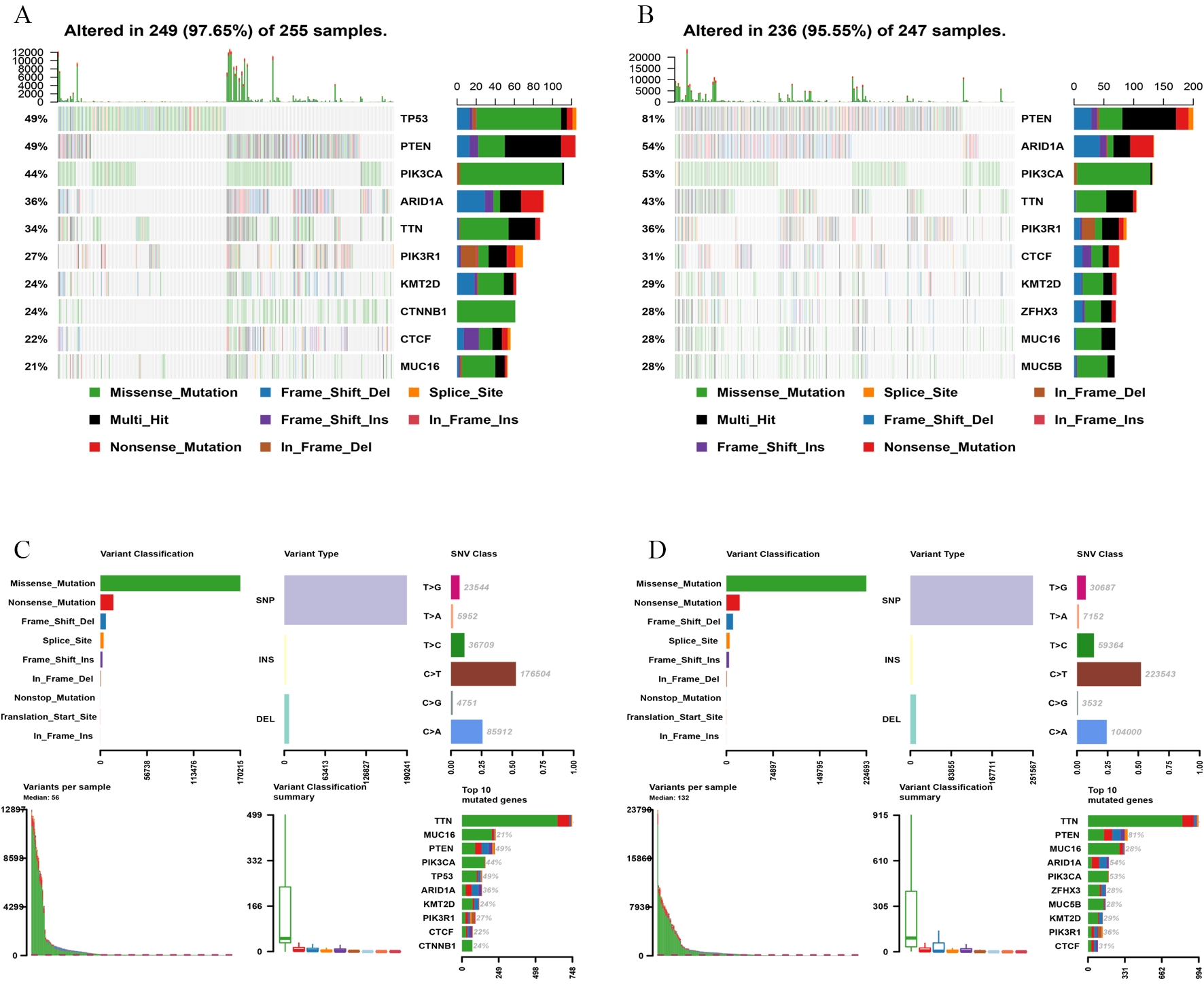

Supplement: Supplementary file 8 — Additional file 8: Figure S7. Somatic mutation analysis. (A) Oncoplot displaying the somatic landscape of EC with high-risk score. (B) Oncoplot displaying the somatic landscape of EC with low-risk score. Stacked bar chart and cohort summary plot displaying distribution of variants according to variant classification, type, and SNV class. Bottompart (from left to right) indicates mutation load for each sample, variant classification type of the high-risk group (C) and low-risk group (D). [file 12935_2020_1264_MOESM8_ESM.tif]
